# Improving rural and remote practitioners’ knowledge of the diabetic foot: findings from an educational intervention

**DOI:** 10.1186/s13047-016-0157-2

**Published:** 2016-07-29

**Authors:** Deborah E. Schoen, Kaniz Gausia, David G. Glance, Sandra C. Thompson

**Affiliations:** 1 Western Australian Centre for Rural Health, Faculty of Medicine, Dentistry & Health Sciences, The University of Western Australia, 35 Stirling Highway, Crawley, WA 6009 Australia; 2 Centre for Software Practice, The University of Western Australia, 35 Stirling Highway, Crawley, WA 6009 Australia

**Keywords:** Diabetic foot, Risk stratification, Knowledge, Attitude, Practice

## Abstract

**Background:**

This study aimed to determine knowledge of national guidelines for diabetic foot assessment and risk stratification by rural and remote healthcare professionals in Western Australia and their implementation in practice. Assessment of diabetic foot knowledge, availability of equipment and delivery of foot care education in a primary healthcare setting at baseline enabled evaluation of the effectiveness of a diabetic foot education and training program for generalist healthcare professionals.

**Methods:**

This study employed a quasi-experimental pre-test/post-test study design. Healthcare practitioners’ knowledge, attitudes and practice of diabetic foot assessment, diabetic foot risks, risk stratification, and use of the 2011 National Health and Medical Research Council Guidelines were investigated with an electronic pre-test survey^.^ Healthcare professionals then undertook a 3-h education and training workshop before completing the electronic post-test knowledge, attitudes and practice survey. Comparison of pre-test/post-test survey findings was used to assess the change in knowledge, attitudes and intended practice due to the workshops.

**Results:**

Two hundred and forty-six healthcare professionals from two rural and remote health regions of Western Australia participated in training workshops. Monofilaments and diabetes foot care education brochures, particularly brochures for Aboriginal people, were reported as not readily available in rural and remote health services. For most participants (58 %), their post-test knowledge score increased significantly from the pre-test score. Use of the Guidelines in clinical settings was low (19 %). The healthcare professionals’ baseline diabetic foot knowledge was adequate to correctly identify the high risk category. However, stratification of the intermediate risk category was poor, even after training.

**Conclusion:**

This study reports the first assessment of Western Australia’s rural and remote health professionals’ knowledge, attitudes and practices regarding the diabetic foot. It shows that without training, generalists’ levels of knowledge concerning the diabetic foot was low and they were unlikely to assess foot risk. The findings from this study in a rural and remote setting cast doubt on the ability of generalist healthcare professionals to stratify risk appropriately, especially for those at intermediate risk, without clinical decision support tools.

**Electronic supplementary material:**

The online version of this article (doi:10.1186/s13047-016-0157-2) contains supplementary material, which is available to authorized users.

## Background

Diabetic foot complications are minimised with good glycaemic control [1, 2] and by prevention, early identification, and management of foot risk factors [3]. Accurate diabetic foot risk stratification predicts foot ulceration [4, 5] and so is a crucial step in the prevention of complications. The 2011 National Health and Medical Research Council (NHMRC) *National Evidence*- *Based Guideline on Prevention*, *Identification and Management of Foot Complications in Diabetes* (Guideline) provided an expert consensus opinion that “[in Australia] any suitably trained health professional may perform the [foot] risk assessment [stratification]” [3]. Unfortunately, in Australia, there is limited evidence available regarding rural and remote healthcare professionals’ diabetic foot knowledge and practice, or on approaches to upskilling health generalists in rural practice about the diabetic foot.

Rural and remote communities in Australia are of particular concern for diabetic foot complications. Rates of diabetes consultations are higher in rural areas than in other areas of Australia [6], rural and remote general practitioners are less confident than their urban collegues managing complications of diabetes [7] and a high proportion of diabetes consultations in very remote [8] Australia occur with Aboriginal patients [6]. There is also evidence that Aboriginal amputees are more likely to reside in a remote community [9]. Diabetes is the second greatest category of expenditure for disease in Aboriginal people, and diabetes complications are the second ranked contributor to potentially preventable hospitalisations in this population [10]. Nearly all, (98 %) of lower extremity amputations in Aboriginal people in Western Australia between 2000 and 2008 were associated with diabetes [11]. Furthermore, parts of Australia, such as Western Australia, have a shortage of podiatrists in rural and remote areas [12, 13]. The Western Australian public health system covers 2.5 million square kilometres and is the largest area in the world covered by a single health authority, so access to specialist podiatry for those living remotely is very difficult, with a strong rationale for the training of generalists to identify foot problems early [14].

While a protocol for a Cochrane systematic review protocol on the education of healthcare professionals around the diabetic foot was published in 2013 [15], the review itself has not been published and no other reviews on this topic were identified. The majority of Cochrane systematic reviews on educating healthcare professionals on various topics include physicians and nurses. However, midwives, dieticians, pharmacists and psychologists have been included in some studies. The reviews have shown continuing professional development [16–18] using mixed interactive and didactic formats [16], printed educational materials [19–22], educational outreach [23], audit and feedback [24, 25] and multifaceted interventions [26] to be consistently effective effective methods of educating healthcare professionals. Randomised controlled diabetes studies that have included diabetes foot care education for healthcare professionals have used printed educational materials [27–32], audit and feedback [28] and multifaceted interventions [27, 28, 30, 32]. These studies resulted in improvement in foot care processes; increased foot examination [27–32], increased appropriate referrals to podiatry [30, 32], increased patient education [30, 32] and increased requests for protective footwear [32, 33]. Diabetic foot studies that have used continuing medical education and a pre-test/post-test study design reported improvement in healthcare professionals’ diabetic foot knowledge [33–35]. Only one diabetic foot study using a pre/post-test study studies and continuing medical education reported improvement in foot care processes and increased requests for protective footwear [33]. Most recently, a non-randomized stepped-wedge design, including a single education session to nurses in a haemodialysis unit combined with patient education resulted in increased foot examinations [36]. Multifaceted interventions including education for healthcare professionals and healthcare systems interventions have the greatest impact on diabetic foot processes that can identify diabetic feet at risk and limit the progression of disease that can ultimately result in ulceration and lower extremity amputation.

This study aimed to determine knowledge of national guidelines for diabetic foot assessment and risk stratification amongst rural and remote healthcare professionals in Western Australia and their implementation in practice. The assessment of diabetic foot knowledge, availability of equipment and delivery of foot care education in a primary healthcare setting at baseline enabled evaluation of the effectiveness of a diabetic foot education and training program for generalist healthcare professionals. (Generalists included doctors, nurses, Aboriginal Health Workers, dieticians, diabetes educators, podiatrists, home and community care workers, students, patient care assistants, therapy assistants, Aboriginal Liaison Officers and Indigenous support officer). The training and education were part of a multifaceted High Risk Foot intervention [37] which included an electronic risk tool with clinical decision support [38], a Multidisciplinary Foot Ulcer Telehealth Clinic, Aboriginal diabetes foot care education brochures [39] and movies [40, 41]. The comprehensive intervention was planned using the World Health Organisation’s Innovative Care for Chronic Conditions Framework [42].

## Methods

### Study design

This study employed a quasi-experimental pre-test/post-test study design. Healthcare practitioners’ knowledge, attitudes and practices (KAP) of diabetic foot risks, assessment procedures, risk stratification, and use of the 2011 NHMRC *Guidelines* were investigated with an electronic pre-test survey [43]. Healthcare professionals then undertook a 3-h mixed interactive and didactic education and training workshop before completing the electronic post-test KAP survey. Comparison of pre-test/post-test KAP survey findings was used to assess the change in knowledge, attitudes and intended practice due to the workshops.

### Study population

Two hundred and forty-six rural and remote healthcare professionals from two country health regions of Western Australia participated in the diabetic foot workshops.

### Setting

Study sites were 15 rural and remote towns in the Midwest and Pilbara regions of Western Australia. Fifteen workshops were delivered in hospitals, five in Aboriginal Community Controlled Health Organisations, five at a rural health centre, two at aged care centres, and one each at a rural university centre, a remote nursing post and rural general practice.

### Sampling

Health service managers or staff development officers disseminated information to staff regarding the workshops by email, through discussion at in-house meetings and by posting workshop flyers on staff development boards around the hospitals and health services. The target group of participants was healthcare practitioners or people involved in the care of people with diabetes at risk of foot disease who could potentially perform a diabetic foot assessment.

### Data collection

Data were collected between June 2012 and June 2013. The pre and post-test KAP survey responses were collected electronically with the TurningPoint® audience response system [43]. This system integrates with Microsoft PowerPoint presentations to allow participants to enter survey responses on handheld keypads. TurningPoint® collects, saves and displays the answers in the Microsoft PowerPoint presentation and into the Microsoft Excel spreadsheet simultaneously.

### Survey instrument

Two surveys were identified from the literature as potential instruments [44, 45], but after review were not considered suitable/specific enough for this study as neither focused on diabetic foot risk assessment and stratification. Hence, for this study, NHMRC *Guidelines* summary recommendations 1, 2, 3, 4 and 7 and expert opinions 1, 3, 4, 5, and 6 which pertain to a primary healthcare setting were translated into question format and piloted for the pre and post-test KAPs [3]. Additional file 1: Changes to Knowledge Attitudes and Practice surveys resulting from piloting.

The pre-test KAP was a 42-item survey covering four content domains: demographics, knowledge, attitudes and practice. Multiple choice and yes/no closed questions were asked for 7 demographic, 16 knowledge and 16 practice questions, and for the 3 attitude questions a five-point Likert scale (1 = strongly agree, 5 = strongly disagree) was used. Additional file 2: Knowledge, Attitude and Practice Survey Pre-test.

The post-test KAP survey consisted of 25 questions: 11 matched knowledge and two matched attitude questions (repeated from the pre-test); two intended practice questions; five unmatched foot deformity questions; and five evaluation questions. Additional file 3: Knowledge, Attitude and Practice Survey Post-test

### The education and training workshops

All workshops commenced with a formal ‘Acknowledgment of Country’ to recognise the specific local Aboriginal ‘tribal’ group as the First Australians and custodians of the land where the workshop was taking place [46]. Workshops were face-to-face, 2 to 3 h in duration, limited to a maximum of 20 participants, and presented by a podiatrist.

The workshop sequence was: pre-test KAP survey; education delivery; hands on training; time for participants to practise a diabetic foot risk assessment using a risk calculator tool with clinical decision support [38]; and at the end of the session, post-test KAP survey. The education component focused on imparting knowledge, raising awareness, and promoting understanding of the diabetic foot. The training included the development of evidence-based foot assessment skills and eHealth communication skills. The workshop was interactive, informal and practical, and based on the adult learning principles that adults learn best informally and by active participation, and that Aboriginal people absorb knowledge better through experiential, hands-on learning [47–49]. The educational resources and training materials supplied to participants and the rationale for providing them are shown in Table 1.

**Table 1:** Education and training materials supplied to participants

Materials	Rationale
Printed educational materials	Education [16, 70]
NHMRC *Guidelines* [3]	Small beneficial effect on HCP practice [19–22, 71]
Western Australian *High Risk Foot Model of Care* [12]	Distribution/passive dissemination of PEM [19–22] Benefit to patient outcome [34, 72, 73]
*The Foot Book*: *A manual for Aboriginal Health Workers about common foot problems*, *how to recognize them and what to do about them* [74]	Culturally appropriate/real photographs of Aboriginal foot complications and foot deformities [75]
Royal Perth Hospital Multidisciplinary Foot Ulcer Telehealth Clinic brochure	Communication/marketing new clinic and educate what should be referred [70]
Royal Perth Hospital Multidisciplinary Foot Ulcer Telehealth Clinic referral form	Promote continuity and coordination of care of diabetic foot [42]
Electronic educational materials on USB	Distribution/passive dissemination of PEM [19–22]
NHMRC *Guidelines* [3]	Electronic materials equivalent effect on HCP practice to PEM [76]
Western Australian *High Risk Foot Model of Care* [12]	EHI and education –HCP used resource more frequently [77]
	Create foot folder on work computer
	Ability to share information with others
	Resource for students and new staff
Diabetic foot diagnostic imaging pathways [78, 79]	Evidence-based pathways in HDWA
Journal articles on the diabetic foot	Distribution/passive dissemination of PEM [19–22]
Images of diabetic foot problems	Facilitate recognition of foot deformities [59, 62]
Royal Perth Hospital Multidisciplinary Foot Ulcer Telehealth Clinic referral form	Promote continuity and coordination of care of diabetic foot [42] Communication/marketing of new clinic [70]
URL links useful for continuing education [80–82]	Access to further CME if interested
	Access to CME for rural and remote HCP
	Online Aboriginal Cultural Orientation Training
For patient assessment	Training [70]
10-g monofilament	Equip teams [42] Enablement [70]
	Enable foot risk stratification
Two different diabetes foot care brochures produced by Diabetes WA [39]	Enable diabetic foot education [3]
	Mobilise resources [42]
	Support self-management [42]
Resources for Aboriginal people from Diabetes WA [39]	Enable culturally appropriate education
	Comply with HDWA guidelines [12, 13, 83, 84]
Education brochures for patients specific to stratified level of foot risk (low, intermediate, high or ulcer care)	Access to education/Education targeted to foot risk

*HCP* Healthcare Professional, *PEM* Printed Education Materials, *USB* Universal Serial Bus, *EHI* Electronic Health Information, *HDWA* Department of Health Western Australia, *CME* Continuing Medical Education, PEMs, defined as the distribution of published or printed recommendations for clinical care including clinical practice guidelines, monographs, and publications in peer-reviewed journals, delivered personally or through mass mailing [85]

### Statistical analysis

Pre and post-test KAP Microsoft Excel data sheets were imported to IBM SPSS Version 22 [50]. The unique identification code of each keypad allowed each participant’s pre and post-test variables to be reliably matched. Negatively worded items in the surveys were reversed. Knowledge answers were recoded to correct or incorrect. Each correct answer was given a score of one point, and incorrect responses scored zero. “Don’t know” responses were considered wrong and scored zero. Total pre and post-test knowledge scores were calculated as the sum of correct responses. A paired *t*-test was used to test the difference between the means of the pre and post-test knowledge scores, as a continuous variable, for all participants with complete pre and post-test knowledge scores. McNemar’s test was used to test the difference between paired proportions for 11-matched knowledge answers and two matched attitude answers. Knowledge answers were only included in McNemar’s test if all 11 knowledge questions were answered. A *p*-value of <0.05 was considered statistically significant, and two-tailed tests were used for significance testing.

Ethics approvals for this study were granted by The University of Western Australia Human Research Ethics Committee (RA/4/1/5054), the Western Australian Aboriginal Health Ethics Committee (363-09/11) and the Western Australian Country Health Service Research Ethics Committee (2011:25).

## Results

### Participant demographics

A total of 246 healthcare professionals from two rural and remote health regions of Western Australia participated in the diabetic foot workshops. Participants were aged between 18 and 65 years; 83 % were female, 14 % identified as Aboriginal and Torres Strait Islander, and half were nurses (51 %). Table 2 displays the demographic information of the workshop participants for the total group attending education (*n* = 246) and those for whom matched pre-test and post-test knowledge scores were available (*n* = 117).

**Table 2:** Demographic characteristics of workshop participants, including the subgroup with matched pre-test and post-test knowledge scores

Demographic	All *n* = 246	(Percent)	Matched *n* = 117	(Percent)
Sex				
Male	25	(10)	10	(8)
Female	204	(83)	106	(91)
Missing	17	(7)	1	(1)
Ethnicity				
Aboriginal and Torres Strait Islander	32	(14)	11	(9)
Non-Aboriginal	193	(78)	104	(89)
Missing	21	(8)	2	(2)
Age (years)				
18–24	17	(7)	12	(10)
25–34	37	(15)	19	(16)
35–44	59	(24)	33	(28)
45–54	65	(26)	34	(30)
55–64	43	(18)	14	(12)
65+	6	(2)	2	(2)
Missing	19	(8)	3	(3)
Job description				
Aboriginal Health Worker	18	(7)	3	(3)
Nurse	125	(51)	68	(58)
General Practitioner	4	(2)	1	(1)
Allied Health	9	(4)	7	(6)
Home and Community Care	11	(4)	6	(5)
Podiatrist	10	(4)	7	(6)
Non-clinical	12	(5)	4	(3)
Other^a^	35	(14)	19	(16)
Missing	22	(9)	2	(2)
Undergraduate training				
Metropolitan Australia	94	(38)	49	(42)
Rural Australia	94	(38)	51	(44)
Overseas	38	(15)	17	(14)
Missing	20	(8)	0	(0)
Duration as health professional (years)				
0–4	48	(19)	27	(23)
5–9	49	(20)	26	(22)
10–14	31	(13)	13	(11)
15–19	28	(11)	16	(14)
20–24	19	(8)	9	(8)
25–29	20	(8)	11	(9)
30+	29	(12)	12	(10)
Missing or Not applicable	22	(9)	3	(3)
Workplace				
Aboriginal Community Controlled Health Organisation	40	(16)	10	(9)
WA Country Health Service	102	(42)	49	(42)
General Practice	20	(8)	18	(15)
Private practitioner (non-medical)	20	(8)	9	(8)
Home and Community Care	6	(2)	4	(3)
Other	41	(17)	25	(21)
Missing	17	(7)	2	(2)
Total	246		117	

^a^Other group includes: students (18), patient care assistants (9), student supervisors/lecturers (3), therapy assistants (2), Aboriginal Liaison Officers (2), Indigenous support officer (1)

### Attitudes

More than 95 % of participants in both pre and post-test believed a foot ulcer is serious, and 83 % believed diabetic foot problems were an issue in their community. Before training, 31 % believed that only a podiatrist can stratify foot risk, and this changed significantly to 19 % (*p* < 0.001) post-training.

### Practice

The availability of resources to complete a diabetic foot assessment is shown in Table 3 Participants’ self-reported foot assessment practice prior to training (*n* = 246). Confidence in doing an assessment increased from 34 % pre-training to 95 % immediately after the intervention (*p* < 0.001). Lack of time was the most frequently reported reason for not checking feet.

**Table 3:** Participants’ reported pre-training practices and foot assessment resources (*n* = 246)

Statements	Percent
Practices	
Regularly check the feet of people with diabetes (*n* = 230)	59
Regularly document when feet of people with diabetes are checked (*n* = 224)	71
Regularly provide foot care education to people with diabetes (*n* = 223)	41
Use the 2011 NHMRC risk stratifications [3] (*n* = 214)	19
Previously trained in foot assessment (*n* = 218)	39
Resources available in clinic	
Diabetes foot care brochures (*n* = 227)	54
Aboriginal diabetes foot care brochures (*n* = 223)	22
10-g monofilament (*n* = 216)	47

### Knowledge

#### Missing values and excluded scores

**Fig. 1:**
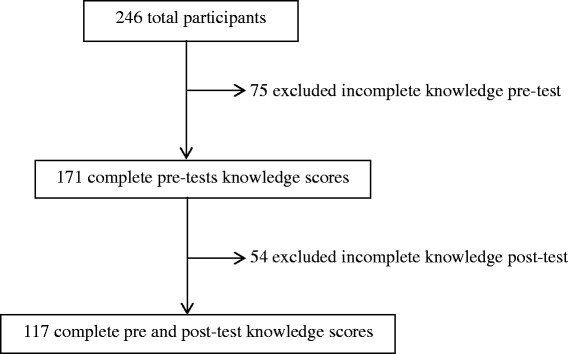
Knowledge scores included in paired pre-test/post-test analysis

Knowledge scores from over half (52.4 %) of workshop participants were excluded from paired data analysis due to missing pre-test or post-test surveys or missing responses to individual items (Fig. 1). There were several reasons for this. A total of 39 participants missed either the entire pre-test (14) or the entire post-test (25), most often due, respectively, to being late to the workshop or needing to return to work before or during the post-test. Knowledge scores for participants (*n* = 20) from five towns (two very remote, one remote and two outer regional) [8] were excluded due to an error using TurningPoint® [43]. Of the remaining 187 participants, 70 randomly missed different questions without any apparent pattern or specific questions missed. Participants who did not respond to questions had similar demographic characteristics to those who answered, except that two-thirds of the knowledge answers of Aboriginal and Torres Strait Islander participants were excluded because paired responses were not available.

#### Changes in knowledge scores among participants providing complete pre-test/post-test data

**Fig. 2:**
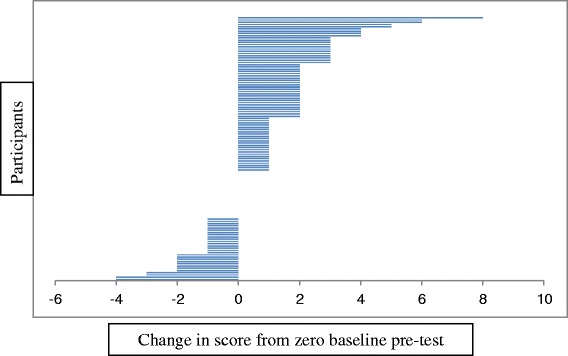
Size and direction of change in knowledge score for each participant

For most participants with paired data, their post-test knowledge score increased from the pre-test score (68/117; 58 %). No change in knowledge score occurred for 18 % (21/117), and 25 % (28/117) had a pre-test score greater than post-test score (Fig. 2). There was no pattern of difference being discernible by any professional group, other than that for Home and Community Care Workers (*n* = 11) where all participants had higher post-test knowledge scores.

The mean knowledge score for the 117 participants was 6.9 ± 1.6 in the pre-test and 7.8 ± 1.4 in the post-test, a significant change in mean knowledge score (0.9, 95 % CI 0.5–1.3) (*p* < 0.001) on a two-tailed paired *t*-test.

#### Individual knowledge questions –paired pre-test /post-test results

Table 4 shows the change in knowledge after training based on individual paired pre-test /post-test results (*n* = 117). The difference between the 117 paired proportions of responses in pre- and post-test showed a significant difference for five of the 11 knowledge questions. Two of the three questions assessing healthcare professionals’ ability to determine people at intermediate risk of diabetic foot complications improved (foot deformity and neuropathy), however, even after the training less than half of the participants could stratify intermediate foot risk correctly. For the question requiring respondents to classify people with non-palpable pulses as being at intermediate risk, there was no improvement with only 32 % answering correctly in the post-test.

**Table 4:** Change in individual knowledge questions –paired pre-test /post-test results (*n* = 117)

Knowledge questions	Pre-test		Post-test		
	Correct *n* (%)	95 % CI	Correct *n* (%)	95 % CI	*p*-value
People with an amputation are high risk^a^	95 (81.2)	74–88	110 (94.0)	90–98.	0.003*
People with a previous foot ulcer are high risk^a^	102 (87.5)	81–93	105 (89.7)	84–95	0.664
People with non-palpable pulses (and no other risk factors or previous history) are intermediate risk ^a^	27 (23.1)	15–31	38 (32.5)	24–41	0.090
People unable to feel monofilament (and no other risk factors or previous history) are intermediate risk^a^	22 (18.8)	12–26	54 (46.2)	37–55	<0.001*
People with a foot deformity (and no other risk factors or previous history) are intermediate risk ^a^	25 (21.4)	14–29	72 (61.5)	53–70	<0.001*
People with two foot risks are high risk (and no previous history of amputation or ulceration)^a^	103 (88.0)	82–94	103 (88.0)	82–94	1.000
Check low risk feet every 12 months^a^	52 (44.4)	35–53	98 (83.8)	77–90	<0.001*
Check high risk feet every 3 months^a^	89 (84.6)	68–84	99 (90.6)	78–91	0.248
Palpate two pulses in each foot	67 (57.3)	48–66	102 (87.2)	81–93	<0.001*

^a^NHMRC evidence-based recommendations; *CI* confidence interval; * statistically significant a *p*-value of < 0.05

#### Five unmatched foot deformity questions

Table 5 shows the results of knowledge questions of all participants pre- and post-test (unpaired). Knowledge of the foot deformities was low in the pre-test and showed the greatest improvement in the intermediate risk category. Improvements in recognition of small muscle wasting increased from 34 % of participants in the pre-test to 85 % in the post-test and knowing how to test for limited joint motion increased from 19 % in the pre-test to 70 % of participants in the post-test. More modest, but still substantial, improvements occurred in participants recognising a hammertoe (from 42 to 60 %) and claw toes (from 52 to 82 %) in pre and post-test assessment.

**Table 5:** Results of knowledge questions^a^

	*n*	Pre-test (%)	*n*	Post-test (%)
Do you know what a hammertoe looks like?	218		213	
Yes	104	42	147	60
Missing	28		33	
Do you know what claw toes look like?	226		201	
Yes	127	52	132	82
Missing	20		45	
Do you know what small muscle wasting looks like?	220		208	
Yes	83	34	164	85
Missing	26		38	
Do you know how to test for limited joint motion?	225		207	
Yes	46	19	172	70
Missing	21		39	

^a^Note: Participants’ responses are not paired

#### Evaluation questions

The workshops were well received based on the post-test evaluation, with most respondents finding the workshop content understandable (85 %), providing useful information (85 %), having appropriate quality and content of information (87 %) and stating they would recommend the workshop to colleagues (87 %).

## Discussion

This study found the preexisiting use of the 2011 NHMRC guidelines was very low, stratification of the intermediate risk category was poor, even after training and the resources to complete a diabetic foot assessment are not readily available in in rural and remote Western Australian health services. Evaluation of the workshops was positive and improved healthcare professionals’ short-term diabetic foot knowledge and confidence to complete a diabetic foot risk stratification.

The pre/post-test design used in this study enabled evaluation of the diabetic foot workshops and allowed self-evaluation by the participants, the optimal method for adult learners to self-assess their progress or success [48, 49, 51]. This approach was appropriate to apply evidence shown to be consistently effective in reducing the gap between research evidence and practice in the education of healthcare professionals [16–18, 20–22, 26, 28, 52–56] and determine the impact of the workshops. Strengths of the study were the use of questions derived from the 2011 NHMRC *Guidelines* using this as a standard based on expert consensus [3] and the inclusion of a broad range of primary healthcare professionals in the workshops reflects the reality of rural and remote practice in Western Australia. The limitation of the pre/post test design is that it reports only on *intended* practice; the effect on actual practice and foot care processes is unknown. Other limitations of this study were the reduced number of matched pre and post-test scores, particularly for the Aboriginal participants and no longer-term follow-up to determine if knowledge was retained.

Other Australian studies reporting higher use of the diabetic foot guidelines sampled podiatrists only, rather than the multidisciplinary sample in this study [57, 58]. Improvement in healthcare practitioners’ diabetic foot knowledge in multidisciplinary samples has been shown in other studies using a pre/post-test design and continuing medical education [33–35]. Most recently a single education session for nurses in a haemodialysis unit resulted in an increased number of foot examinations [36]. The inability of health professionals to correctly stratify diabetic foot risk, even after training, has been reported previously in an urban setting in Western Australia [59]. Neither an increase in foot care processes as in other studies [27–32, 36], nor assessment of patient outcomes as reported by others [7, 60, 61] can be determined from this study.

The clinical implication of the findings that healthcare professionals cannot identify foot deformities aligns with those reported by others [30, 59, 62]. Foot deformity was excluded from the PODUS study [5] as it was not consistently defined in the included data sets, yet it appears in most diabetic foot risk stratification systems worldwide [3, 63–66]. The qualitative nature of foot deformity limits it’s inclusion in research protocols despite it’s recognised clinical importance as a risk factor for diabetic foot ulceration. This study supports the NHMRC expert consensus that health professionals in Australia need to be *suitably trained* to perform diabetic foot risk stratification [3]. An Australian diabetic foot education programme such as the interactive online diabetic foot training offered by the Scottish Diabetes Group and University of Edinburgh’s Foot Risk Awareness and Management Education (FRAME) program is warranted to support healthcare professionals to develop and maintain the necessary skills and competencies in diabetic foot examination [67].

The question of whether or not generalists can competently complete foot risk stratification is still unresolved from this study. Alternative approaches to education and training such as the FRAME program [67] or telehealth education, now offered by Diabetes WA in Western Australia need to be evaluated [68]. Similarly, the Indigenous Diabetic Foot Program, a 2-day program particularly for Aboriginal and Torres Strait Islander peoples to encourage diabetic foot assessment, requires evaluation [69].

## Conclusion

This study reports the first assessment of Western Australia’s rural and remote health professionals’ knowledge, attitudes and practices regarding the diabetic foot. It shows that without training, generalists’ levels of knowledge concerning the diabetic foot was low and they were unlikely to assess foot risk. The findings from this study in a rural and remote setting cast doubt on the ability of generalist healthcare professionals to stratify risk appropriately, especially for those at intermediate risk, without clinical decision support tools. Alternative approaches to training which reliably improve knowledge and support consolidation of learning and translation into practice over the longer term need to be trialled and assessed.

## Abbreviations

CI, Confidence interval; CME, Continuing Medical Education; EHI, Electronic Health Information; FRAME, Foot Risk Awareness and Management Education; HDWA, Department of Health Western Australia; HCP, Healthcare Professional; KAP, Knowledge, attitudes and practices; NHMRC, National Health and Medical Research Council; PEM, Printed Education Materials; USB, Universal Serial Bus; WA, Western Australia

## Additional files

Additional file 1: Knowledge, Attitudes and Practice Survey resulting from piloting. (DOCX 24 kb) Additional file 2: Knowledge, Attitude and Practice Survey Pre-test. (DOCX 30 kb) Additional file 3: Knowledge, Attitude and Practice Survey Post-test. (DOCX 1821 kb)
