# Supplementary material for: Improving rural and remote practitioners’ knowledge of the diabetic foot: findings from an educational intervention
Source: J Foot Ankle Res. 2016 Jul 29;9:26. doi: 10.1186/s13047-016-0157-2 (PMC4966728; doi:10.1186/s13047-016-0157-2)
Supplement: Additional file 1: — Knowledge, Attitudes and Practice Survey resulting from piloting. (DOCX 24 kb) [file 13047_2016_157_MOESM1_ESM.docx]

Changes to Knowledge Attitudes and Practices Assessment resulting from piloting

| **Practical tips** |  |
| --- | --- |
| Tell future participants to look at the keypad rather than at the PowerPoint when inputting answer into keypad. | Included in introduction to workshops. |
| Tell participants to press the number keys firmly. | Included in introduction to workshops. |
| **Errors** |  |
| Slide regarding how many sites are tested for the monofilament there was an option for 10 but no number ten on the keypads. | Multiple choices altered to options 1-9, 9 or 10 sites tested combined as option 9. |
| Error in University of Texas Diabetic Foot Risk Classification System**;** had 1-6. | Slide corrected to show Texas Diabetic Foot Risk Classification System 0-6. |
| Question regarding infection ambiguous. | Question removed as no definite answer from National Health and Medical Research Council Guideline [1]. |
| **Suggestions** |  |
| Couldn’t see the numbers on the side of the PowerPoint well enough. | Numbers put in a different colour. |
| Monofilament question, from “Do you have a monofilament?” not clear. | Added “in your practice”  “Do you have a monofilament in your practice?” |
| Incomplete choices for where education completed. | Added rural Australia as a choice for where education completed. |
| Showing results for age slide not appropriate. | Do not show the results for the age slide. |
| Wording of question about how many sites people do not feel to be insensate. | How many sites do people have to NOT feel with the monofilament to be at risk? |
| Foot deformity questions provided inadequate choices. | Added don’t know /not sure answer options. |
| **Did not understand** |  |
| What do you mean by “Diabetic foot problems are a serious problem in the community”? | Added “my” community to question.  “Diabetic foot problems are a serious problem in my community” |
| Not everyone understood Word “stratify” in question asking about diabetic foot risk system used. | Wording of question changed to “classify” not stratify. |
| What do you mean by “the system to stratify foot risk”? | Reworded as two questions:  1) Do you use a foot risk classification system?  2) Which foot risk classification system do you use? |

**References**

1. National Health and Medical Research Council. National Evidence-Based Guideline: Prevention, Identification and Management of Foot Complications in Diabetes. (Part of the Guidelines on the management of Type 2 Diabetes). National Health and Medical Research Council, Melbourne. 2011. <http://t2dgr.bakeridi.edu.au/LinkClick.aspx?fileticket=anrL23t3ADw%3d&tabid=172>. Accessed 10 Apr 2011.
