# Supplementary material for: Improving rural and remote practitioners’ knowledge of the diabetic foot: findings from an educational intervention
Source: J Foot Ankle Res. 2016 Jul 29;9:26. doi: 10.1186/s13047-016-0157-2 (PMC4966728; doi:10.1186/s13047-016-0157-2)
Supplement: Additional file 2: — Knowledge, Attitude and Practice Survey Pre-test. (DOCX 30 kb) [file 13047_2016_157_MOESM2_ESM.docx]

### Additional file 2: Knowledge, Attitude and Practice Survey Pre-test

1. Icebreaker
2. Icebreaker
3. Where do you work?

| 1. Aboriginal Medical Service |
| --- |
| 1. WA Country Health Service |
| 1. GP clinic |
| 1. Private practitioner |
| 1. Home and Community Care |
| 1. Medicare local |
| 1. Other |

1. What sex are you?

| 1. Male |
| --- |
| 1. Female |

1. Are you Aboriginal or Torres Strait Islander?

| 1. Aboriginal |
| --- |
| 1. Torres Strait Islander |
| 1. Aboriginal Torres Strait Islander |
| 1. No |

1. What is your primary job role?

| 1. Aboriginal Health Worker |
| --- |
| 1. Nurse |
| 1. Doctor |
| 1. Allied Health |
| 1. Home & Community Care |
| 1. Podiatrist |
| 1. Non-clinical |
| 1. Other |

1. What age bracket do you fit into?

| 1. 18-24 |
| --- |
| 1. 25-34 |
| 1. 35-44 |
| 1. 45-54 |
| 1. 55-64 |
| 1. 65+ |

1. How many years have you worked in Health?

| 1. 0-4 |
| --- |
| 1. 5-9 |
| 1. 10-14 |
| 1. 15-19 |
| 1. 20-24 |
| 1. 25-29 |
| 1. 30+ |

1. Did you receive your training in health in

| 1. Metro Australia |
| --- |
| 1. Rural Australia |
| 1. Overseas |

1. Diabetic foot problems are a serious problem in my community. Do you?

| 1. Strongly Agree |
| --- |
| 1. Agree |
| 1. Neutral |
| 1. Disagree |
| 1. Strongly Disagree |

1. Only a podiatrist can assess feet properly…..do you

| 1. Strongly Agree |
| --- |
| 1. Agree |
| 1. Neutral |
| 1. Disagree |
| 1. Strongly Disagree |

1. How many pulses do you palpate in each foot?

| 1. One |
| --- |
| 1. Two |
| 1. Three |
| 1. Four |
| 1. Don’t know |

1. A foot ulcer is serious.

| 1. Strongly Agree |
| --- |
| 1. Agree |
| 1. Neutral |
| 1. Disagree |
| 1. Strongly Disagree |

1. A foot ulcer is best left open for the air to get to it.

| 1. True |
| --- |
| 1. False |
| 1. Don’t know |

1. I check the feet of people with diabetes

| 1. Always |
| --- |
| 1. Mostly |
| 1. Sometimes |
| 1. Rarely |
| 1. Never |

1. Do you have diabetes foot care education brochures in your clinic?

| 1. Yes |
| --- |
| 1. No |
| 1. Not sure |

1. Do you have Aboriginal diabetes foot care education brochures in your clinic?

| 1. Yes |
| --- |
| 1. No |
| 1. Not sure |

1. I provide foot care education to people with diabetes

| 1. Always |
| --- |
| 1. Mostly |
| 1. Sometimes |
| 1. Rarely |
| 1. Never |

1. People with diabetes may not feel minor injuries to their feet.

| 1. Strongly Agree |
| --- |
| 1. Agree |
| 1. Neutral |
| 1. Disagree |
| 1. Strongly Disagree |

1. Do you have a monofilament in your practise?

| 1. Yes |
| --- |
| 1. No |
| 1. Not sure |

1. Have you ever used a monofilament?

| 1. Yes |
| --- |
| 1. No |

1. How many sites do you test with a monofilament on each foot?

| 1. One |
| --- |
| 1. Two |
| 1. Three |
| 1. Four |
| 1. Five |
| 1. Six |
| 1. Seven |
| 1. Eight |
| 1. Nine or ten |
|  |

1. How many sites do people have to NOT feel with the monofilament to be at risk?

| 1. One |
| --- |
| 1. Two |
| 1. Three |
| 1. Four |
| 1. Five |
| 1. Six |
| 1. Seven |
| 1. Eight |
| 1. Nine or ten |
|  |

1. Do you do any other tests for sensation in the feet?

| 1. Yes 2. No |
| --- |
| 1. Not sure |

1. I document when I have checked people’s feet

| 1. Always |
| --- |
| 1. Mostly |
| 1. Sometimes |
| 1. Rarely |
| 1. Never |

1. Do you know what a hammertoe looks like?
2. Yes
3. No
4. Maybe
5. Do you know what claw toes look like?

| 1. Yes |
| --- |
| 1. No |
| 1. Maybe |

1. Do you know what small muscle wasting looks like?

| 1. Yes |
| --- |
| 1. No |
| 1. Maybe |

1. Do you know how to test for limited joint motion?

| 1. Yes |
| --- |
| 1. No |
| 1. Maybe |

1. Do you know what a Charcot foot looks like?

| 1. Yes |
| --- |
| 1. No |
| 1. Maybe |

1. Do you use a system to classify people’s foot risk?

| 1. Yes |
| --- |
| 1. No |
| 1. Not sure |

1. What system do you use to classify people’s foot risk?

| 1. Texas ( Category 0-6) |
| --- |
| 1. Indigenous Diabetic Foot (Low/High) |
| 1. 2011 NHMRC (low/intermediate/high) |
| 1. Other |
| 1. Don’t know |

1. What level of risk is a person with a foot ulcer?

| 1. Low |
| --- |
| 1. Intermediate |
| 1. High |
| 1. Don’t know |

1. What level of risk is a person with an amputation?

| 1. Low |
| --- |
| 1. Intermediate |
| 1. High |
| 1. Don’t know |

1. What level of risk is a person with pulses you cannot feel only?

| 1. Low |
| --- |
| 1. Intermediate |
| 1. High |
| 1. Don’t know |

1. What level of foot risk is a person when they can NOT feel the monofilament only?

| 1. Low |
| --- |
| 1. Intermediate |
| 1. High |
| 1. Don’t know |

1. What level of foot risk is a person when they have a foot that won’t fit into normal shoes only?

| 1. Low |
| --- |
| 1. Intermediate |
| 1. High |
| 1. Don’t know |

1. What level of foot risk is a person if when they have a foot that won’t fit into normal shoes and can NOT feel the monofilament?

| 1. Low |
| --- |
| 1. Intermediate |
| 1. High |
| 1. Don’t know |

1. Have ever been shown how to do a foot assessment?

| 1. Yes |
| --- |
| 1. No |

1. Who most recently trained you to do a foot assessment?

| 1. WoundsWest |
| --- |
| 1. Indigenous Diabetic Foot Program |
| 1. Undergraduate training |
| 1. Current workplace |
| 1. A Podiatrist |
| 1. Continuing education |
| 1. Other |
| 1. Not applicable |

1. How confident are you in doing a foot assessment?

| 1. Very confident |
| --- |
| 1. Confident |
| 1. Not confident |
| 1. Never done one |

1. A low risk person should have their feet checked every…..

| 1. 3 months |
| --- |
| 1. 6 months |
| 1. 9 months |
| 1. 12 months |
| 1. Not sure |

1. A high risk person should have their feet checked every…..

| 1. 3 months |
| --- |
| 1. 6 months |
| 1. 9 months |
| 1. 12 months |
| 1. Not sure |

1. What kinds of things stop you from checking feet?

| 1. Smelly |
| --- |
| 1. Dirty |
| 1. Don’t like feet |
| 1. Not my job |
| 1. Not enough time |
| 1. We have a podiatrist |
| 1. Other |
