# Supplementary material for: Improving rural and remote practitioners’ knowledge of the diabetic foot: findings from an educational intervention
Source: J Foot Ankle Res. 2016 Jul 29;9:26. doi: 10.1186/s13047-016-0157-2 (PMC4966728; doi:10.1186/s13047-016-0157-2)
Supplement: Additional file 3: — Knowledge, Attitude and Practice Survey Post-test. (DOCX 1821 kb) [file 13047_2016_157_MOESM3_ESM.docx]

### Additional file 3: Knowledge, Attitude and Practice Survey Post-test

1. What is your primary job role?

| 1. Aboriginal Health Worker |
| --- |
| 1. Nurse |
| 1. Doctor |
| 1. Allied Health |
| 1. Home & Community Care |
| 1. Podiatrist |
| 1. Non-clinical |
| 1. Other |

1. Only a podiatrist can stratify foot properly…..do you
2. Strongly Agree
3. Agree
4. Neutral
5. Disagree
6. Strongly Disagree
7. A foot ulcer is serious.
8. Strongly Agree
9. Agree
10. Neutral
11. Disagree
12. Strongly Disagree
13. How many pulses should you palpate in each foot?
14. One
15. Two
16. Three
17. Four
18. Don’t know
19. A foot ulcer is best left open for the air to get to it.
20. True
21. False
22. Don’t know
23. How many sites will you test with a monofilament on each foot?
24. One
25. Two
26. Three
27. Four
28. Five
29. Six
30. Seven
31. Eight
32. Nine or ten
33. How many sites will people have to NOT feel with the monofilament to be at risk?
34. One
35. Two
36. Three
37. Four
38. Five
39. Six
40. Seven
41. Eight
42. Nine or ten
43. Name one foot deformity on these feet


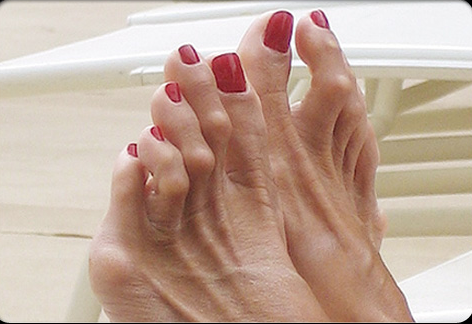


1. Hammertoe
2. Claw toe
3. Small muscle wasting
4. Bony Prominence
5. Name one foot deformity on these feet


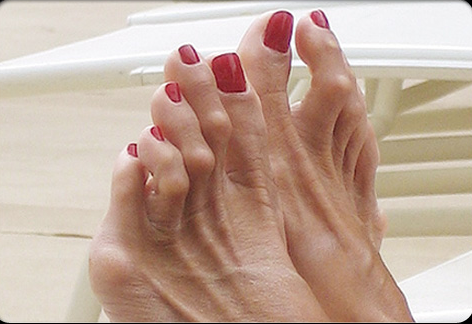


1. Hammertoe
2. Claw toe
3. Small muscle wasting
4. Would sort of toe would this be?


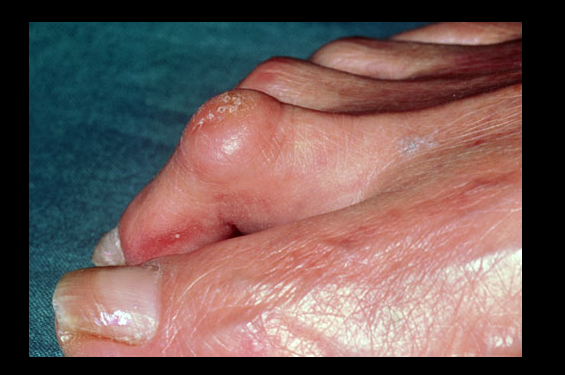


1. Hammertoe
2. Claw toe
3. Mallet toe
4. Name one foot deformity on this foot


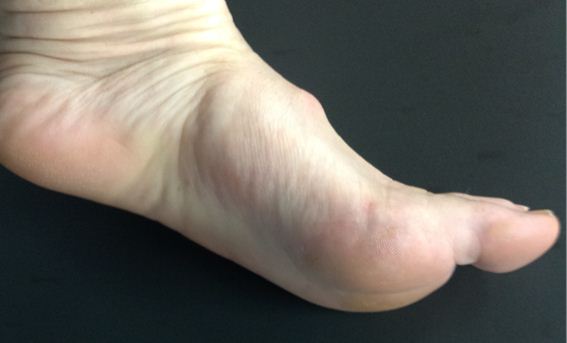


1. Bony prominence
2. Charcot foot
3. Prominent metatarsals
4. Would this be limited joint motion?

1. Yes
2. No
3. What level of risk is a person with a foot ulcer?
4. Low
5. Intermediate
6. High
7. Don’t know
8. What level of risk is a person with an amputation?
9. Low
10. Intermediate
11. High
12. Don’t know
13. What level of risk is a person with pulses you cannot feel?
14. Low
15. Intermediate
16. High
17. Don’t know
18. What level of foot risk is a person when they cannot feel the monofilament?
19. Low
20. Intermediate
21. High
22. Don’t know
23. What level of foot risk is a person when they have a foot that won’t fit into normal shoes?
24. Low
25. Intermediate
26. High
27. Don’t know
28. What level of foot risk is a person if: their foot that won’t fit into normal shoes and they cannot feel the monofilament?
29. Low
30. Intermediate
31. High
32. Don’t know
33. A low risk person should have their feet checked every…..
34. 3 months
35. 6 months
36. 9 months
37. 12 months
38. Not sure
39. A high risk person should have their feet checked every…..
40. 3 months
41. 6 months
42. 9 months
43. 12 months
44. Not sure
45. How confident are you in doing a foot assessment?
46. Very confident
47. Confident
48. Not confident
49. Never done one
50. Overall, this session was understandable to me.

| 1. Strongly Agree |
| --- |
| 1. Agree |
| 1. Disagree |
| 1. Strongly Disagree 2. Don’t want to say |

1. Overall, this session was provided useful information to me.

| - - 1. Strongly Agree |
| --- |
| - - 1. Agree |
| - - 1. Disagree |
| - - 1. Strongly Disagree     2. Don’t want to say |

1. Overall, the quality and content of the information was appropriate to me.

| 1. Strongly Agree |
| --- |
| 1. Agree |
| 1. Disagree |
| 1. Strongly Disagree 2. Don’t want to say |

1. Would you recommend this workshop to friends or colleagues?

| 1. Strongly Agree |
| --- |
| 1. Agree |
| 1. Disagree |
| 1. Strongly Disagree 2. Don’t want to say |

1. Would you like to have the Keypad system at future sessions?

| 1. Strongly Agree |
| --- |
| 1. Agree |
| 1. Disagree |
| 1. Strongly Disagree 2. Don’t want to say |

1. Would you like to use simulated models again at future sessions? **

| 1. Strongly Agree |
| --- |
| 1. Agree |
| 1. Disagree |
| 1. Strongly Disagree 2. Don’t want to say |

1. The simulation exercise enhanced your learning experience? **

| 1. Strongly Agree |
| --- |
| 1. Agree |
| 1. Disagree |
| 1. Strongly Disagree 2. Don’t want to say |

1. The simulation exercise helps you develop practical clinical skills. **

| 1. Strongly Agree |
| --- |
| 1. Agree |
| 1. Disagree |
| 1. Strongly Disagree 2. Don’t want to say |

** Asked for simulation workshops only
